# Polydopamine-encapsulated modified Pulsatilla decoction: a strategy to enhance ulcerative colitis therapy

**DOI:** 10.1186/s13020-025-01280-1

**Published:** 2026-01-05

**Authors:** Ying-Jian Chen, Cheng-Qi Li, Chang Liu, Yi-Jun Zhu, Jing-Jing Wu, Ting-Ting Wu, Hui-Ping Zhu, Dao-Ben Hua, Hong-Wen Sun

**Affiliations:** 1 Department of Internal Medicine, Suzhou TCM Hospital Affiliated to Nanjing University of Chinese Medicine, Suzhou, 215003 China; 2 State Key Laboratory of Radiation Medicine and Protection, School for Radiological and Interdisciplinary Sciences (RAD-X), Soochow University, Suzhou, 215123 China

**Keywords:** Ulcerative colitis, Pulsatilla, Pulsatilla decoction, Polydopamine, Nanoparticles, Tight junction

## Abstract

**Background:**

In Traditional Chinese Medicine (TCM), ulcerative colitis (UC) is often categorized as "protracted dysentery." Pulsatilla Decoction has been reported to exert therapeutic benefits in patients with protracted dysentery. To potentially improve therapeutic outcomes in ulcerative colitis, we prepared a modified Pulsatilla Decoction (MPD). Preliminary clinical observations have suggested that MPD may alleviate symptoms in UC patients. However, rectal enema of MPD is often limited by suboptimal patient compliance and relatively short colonic retention. These limitations underscore the need for new TCM formulations. In this study, we encapsulated MPD within polydopamine (PDA) nanoparticles to prolong colonic residence and evaluate therapeutic effects in a UC model.

**Methods:**

First, PDA@MPD was prepared by encapsulating MPD with PDA. Fourier transform infrared spectroscopy (FT-IR) and other analytical instruments characterized its structure, morphology, and particle size. Drug release property was evaluated by UV–vis spectrophotometry. Subsequently, MPD active components were labeled with Fluorescein isothiocyanate (FITC); PDA@FITC-MPD was prepared similarly and administered orally to mice. In vivo fluorescence imaging tracked retention time and location in the gastrointestinal tract. Finally, the UC model was induced with 3% DSS. After 7 days of PDA@MPD treatment, therapeutic efficacy was assessed via disease activity index (DAI), colon length, histopathology, Western blot, quantitative real-time PCR (qRT-PCR), and ELISA.

**Results:**

MPD was encapsulated by PDA. Relative to MPD, PDA@MPD showed a prolonged colonic retention time and modest improvements in colonic damage scores and inflammatory markers in DSS-induced UC mice. These changes were associated with up-regulation of tight-junction proteins (Occludin, ZO-1) and down-regulation of pro-inflammatory cytokines (IL-1β, TNF-α) in the PDA@MPD group.

**Conclusion:**

This study indicates that PDA@MPD prolongs colonic retention and is associated with modest improvements in DSS-induced colonic damage and inflammation in mice. These findings may offer a proof-of-concept for exploring polydopamine-based delivery systems in TCM formulations.

**Graphical Abstract:**

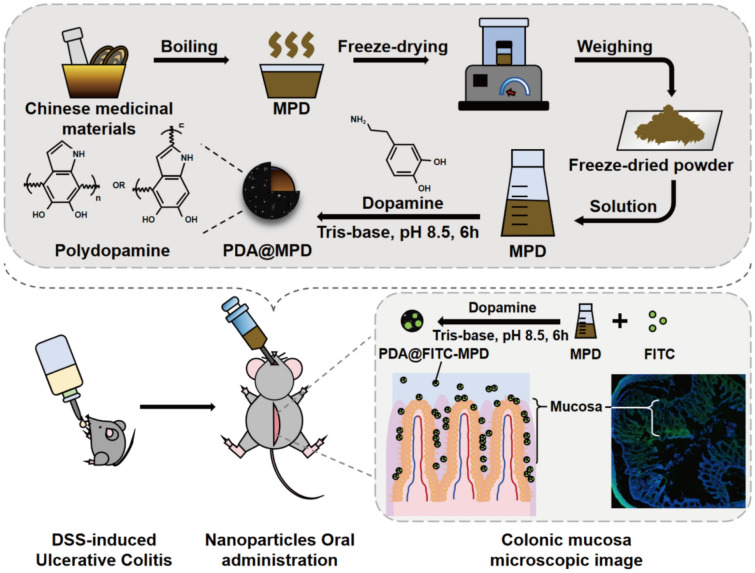

**Supplementary Information:**

The online version contains supplementary material available at 10.1186/s13020-025-01280-1.

## Highlights

1. Polydopamine (PDA) was used to encapsulate the modified Pulsatilla decoction (MPD), yielding PDA@MPD composite nanoparticles.
2. PDA@MPD showed a modestly prolonged retention time on colonic mucosa compared with MPD alone.
3. PDA@MPD alleviated DSS-induced colonic damage and reduced intestinal inflammation to a greater extent than unmodified MPD.

**Supplementary Information:**

The online version contains supplementary material available at 10.1186/s13020-025-01280-1.

## Introduction

Ulcerative colitis (UC) is a type of inflammatory bowel disease (IBD), characterized primarily by symptoms such as diarrhea, intestinal bleeding, and abdominal pain. The global incidence of UC is rising annually, posing increasing healthcare challenges. In Western Europe and North America, pooled incidence rates range from 10.6 to 29.3 cases per 100,000 person-years. A nationwide survey estimated approximately 350,000 IBD cases in China during 2005–2014, and modelling studies project that the number may reach 1.5 million by 2025 [1–3]. Over the past few decades, UC's high incidence and associated disabilities have substantially increased societal treatment and care costs, highlighting the need for effective preventive strategies. Although the precise aetiology of UC remains elusive, available evidence indicates that dysregulation of mucosal immune homeostasis and compromise of the intestinal epithelial barrier contribute to disease initiation [4]. First-line pharmacotherapy for UC includes aminosalicylates, corticosteroids and immunosuppressants. However, these medications may have drawbacks, including limited efficacy and adverse effects [5]. Therefore, identifying new therapeutic options for UC remains an active area of research.

The classical Chinese text *On Typhoid and Miscellaneous Diseases* states that Pulsatilla Decoction "is indicated for heat-type dysentery accompanied by tenesmus." Pulsatilla Decoction primarily consists of four traditional Chinese medicinal herbs: *Pulsatilla chinensis* (Baitouweng), *Coptis chinensis* (Huanglian), *Phellodendron amurense* (Huangbai), and *Fraxinus rhynchophylla* (Qinpi). Its main active chemical components include Anemoside B4 (AB4), Luteolin, Berberine, and Fraxetin. Pharmacological studies of these compounds have shown anti-inflammatory, antioxidant, antibacterial and wound-healing effects in vitro and in animal models, suggesting they may contribute to UC management [6–9]. The Department of Gastroenterology, Suzhou Traditional Chinese Medicine Hospital, developed a modified Pulsatilla Decoction (MPD) based on the academic tradition of the Wu Men Medical School. MPD is prepared by supplementing the original formula with *Panax notoginseng* (Sanqi), *Bletilla striata* (Baiji), *Agrimonia pilosa* (Xianhecao) and *Lithospermum erythrorhizon* (Zicao). Preliminary clinical observations suggested that MPD enema was associated with reduced inflammatory and oxidative stress markers and serum endotoxin, and altered intestinal microcirculation and microbiota composition. These observations suggest MPD may support mucosal barrier repair and clinical symptom relief in UC patients [10].

To achieve local colonic drug levels, MPD is currently administered as a retention enema, which delivers the formulation directly to the colonic mucosa. This modality is limited by suboptimal patient compliance, the need for trained personnel, and relatively short colonic retention of the solution. Therefore, these limitations underscore the need for alternative dosage forms of traditional Chinese medicine.

Polydopamine (PDA), a mussel-inspired polymer, has attracted interest for surface modification due to its high self-polymerization ability under alkaline conditions and its ability to deposit on various materials [11]. PDA possesses a range of distinct physicochemical properties that can impart specific functions to nanoparticles with specific functions. For example, its extraordinary adhesion to organic or inorganic substrates, acid resistance, and good biocompatibility have been extensively studied in the medical field, endowing nanoparticles with potential acid tolerance in the stomach and water-resistant adhesion in the intestine [12–16]. PDA layers can be further functionalised for targeting, offer pH-dependent drug release and show photothermal conversion capacity. Given PDA’s pH-dependent release profile and the near-neutral pH of the colon, we hypothesise that PDA-encapsulated MPD may release a substantial fraction of its active constituents close to the colonic mucosa, potentially improving local drug levels [17]. Thus, the present work provides a proof-of-concept for a PDA-based TCM delivery system in UC and could offer insights for similar approaches to other intestinal disorders.

## Methods

### Materials and reagents

MPD (Pulsatilla, Coptis chinensis, Phellodendron amurense, Cortex Fraxini, Notoginseng, Bletilla striata, Agrimonia Pilosa, Lithospermum erythrorhizon) was purchased from the Suzhou TCM Hospital Affiliated to Nanjing University of Chinese Medicine (China). The Chinese medicine monomers Anemoside B4 (CAS No.: 129741–57-7), Ginsenoside RB1 (CAS No.: 41753–43-9), and Militarine (CAS No.: 58139–23-4) were purchased from Shanghai Shifeng Biotechnology Co., Ltd (China). Trizma® Base and 3-Hydroxytyramine Hydrochloride (Dopamine Hydrochloride) was sourced from Sigma-Aldrich (St. Louis, MO, USA). Fluorescein Isothiocyanate (CAS No.: 3326–32-7) was purchased from MedChemExpress LLC (China). Dextran Sulfate Sodium Salt (DSS, 36,000–50,000 Da, CAS No.: 9011–18-1) was obtained from MP Biomedicals (China). Antifade Mounting Medium with DAPI was purchased from Beyotime Biotechnology (China). Hematoxylin and Eosin Staining Kit was obtained from Beyotime Biotechnology (China). Occult Blood (OB) Reagent was sourced from BASO (China). Anti-β-Actin Mouse Monoclonal Antibody (CAS No.: KGC6106-1), Goat anti-Rabbit IgG Secondary Antibody HRP Conjugated (CAS No.: KGC6202-0.1), Goat anti-Mouse IgG Secondary Antibody HRP Conjugated (CAS No.: KGC6203-0.1), Primary Antibody Dilution Buffer, Total Protein Extraction Kit, SDS-PAGE Gel Preparation Kit, ECL Detection Kit, 5 × SDS-PAGE Protein Loading Buffer, PVDF Membrane, Mouse interleukin-1 beta (IL-1β) ELISA Kit, Mouse interleukin-10 (IL-10) ELISA Kit, Mouse Tumor Necrosis Factor alpha (TNF-α) ELISA Kit were purchased from Kaiji Biotechnology Co., Ltd. (China). IL-1β Polyclonal Antibody (Cat No. 16806–1-AP, Lot: 00139661), Occludin Polyclonal Antibody (Cat No. 27260–1-AP, Lot: 00120869), GAPDH Monoclonal antibody (Cat No. 60004–1-Ig, Lot: 10,021,642) were obtained from Wuhan Sanying Biotechnology Co., Ltd. TNF-alpha Polyclonal Antibody (Cat No. YM8306, Lot: RX423) were obtained from Immunoway Biotechnology Co., Ltd. Anti-β-Actin (Cat No. KGC6106-1, Lot: 20,240,524) were obtained from Kaiji Biotechnology Co., Ltd. Tag Pro Universal SYBR qPCR Master Mix (CAS No.: Q712-02), Hiscript III RT SuperMix for qPCR (+ gDNA wiper) (CAS No.: R323-01) were purchased from Nanjing Novozyme Biotechnology Co., Ltd.

### Preparation of MPD

MPD (*Pulsatilla chinensis* 30 g, *Fraxinus rhynchophylla* 30 g, *Phellodendron amurense* 10 g, *Coptis chinensis* 10 g, *Agrimonia pilosa* 30 g, *Panax notoginseng* 6 g, *Lithospermum erythrorhizon* 30 g, *Bletilla striata* 20 g) was soaked in 450 mL of distilled water for 30 min and then decocted and filtered twice. The resulting filtrate was concentrated to a final volume of 45 mL under vacuum and then freeze-dried to obtain the MPD lyophilized powder.

### Synthesis of PDA@MPD

The PDA self-polymerized nanoparticles were synthesized according to the literature method [18]. One hundred milligrams of MPD lyophilized powder were dissolved in 0.01 M Tris buffer, and dopamine hydrochloride (2.0 mg mL^−1^) was added. The solution was left undisturbed until it gradually turned dark brown. Ethyl acetate was used as the oil phase, and Tween was used as the emulsifier. The aqueous phase was added dropwise to the oil phase to prepare a water-in-oil (W/O) emulsion, which was then added to the emulsifier-containing aqueous phase. After rotary evaporation and freeze-drying, the black product, PDA@MPD, was obtained.

### FITC labeling of PDA@MPD

One hundred milligrams of MPD lyophilized powder was dissolved in 1 mL of distilled water, and the pH was adjusted to 8.5 with 0.5 mol L^−1^ Na₂CO₃. Fifty milligrams of FITC was added, and the mixture was reacted in the dark at room temperature for 24 h. Anhydrous ethanol was then added to the solution to four times the volume, resulting in a yellow-green precipitate. The precipitate was separated by centrifugation, redissolved in water, and reprecipitated with anhydrous ethanol. This process was repeated three times. The supernatant was analyzed using a fluorescence spectrophotometer until no FITC absorption was detected at the specified wavelength. The resulting precipitate was freeze-dried to obtain the FITC-MPD product, which was stored in the dark. The fluorescently labeled drug was dissolved in PBS and subjected to UV–visible spectroscopy scanning in the wavelength range of 200–900 nm to observe the absorption curve at 490 nm and confirm successful fluorescence labeling. After successful labeling, PDA@FITC-MPD and PDA@FITC-AB4 were synthesized according to the method described in the preceding section.

### PDA@MPD characterization

The chemical structure of PDA@MPD was analyzed with a Thermo Scientific Nicolet iN10 Fourier Transform Infrared Spectrometer. The morphology and particle size distribution of PDA@MPD were characterized using a Tecnai G2 F20 S-Twin transmission electron microscope (FEI, USA). The hydrodynamic particle size and surface charge of PDA@MPD were analyzed using a Malvern Zetasizer Model-Nano ZS (Malvern Instruments, UK).

### Animals and experiment protocol

Male C57BL/6 mice weighing 20–22 g were purchased from Sinocell Technology Co., Ltd. (Suzhou, China). All animal care and experimental procedures in this study were approved by the Ethics Committee of the Suzhou TCM Hospital Affiliated to Nanjing University of Chinese Medicine (Animal Ethics Committee Approval No. 2024-LDP-046). All mice were housed in an SPF-grade animal facility at the Experimental Animal Center with a humidity of 55% ± 2%, a temperature of 22 ± 2 °C, and a 12-h light/dark cycle. All procedures strictly followed the National Institutes of Health Guidelines for Care and Use of Laboratory Animals.

First Experimental Group: Mice were randomly divided into four groups: Control group (label-free PDA@MPD, 3.12 g kg^−1^), FITC-MPD group (3.12 g kg^−1^), PDA@FITC-MPD group (3.90 g kg^−1^), and PDA@FITC-AB4 group (100 mg kg⁻^1^). All groups were administered the corresponding drugs via oral gavage. Mice were anesthetized with sodium pentobarbital (150 mg kg^−1^, i.p.), euthanized by cervical dislocation, and the entire gastrointestinal tract was immediately excised. The intestinal distribution of the drugs was observed by detecting FITC fluorescence intensity at 2 h, 6 h, and 24 h.

Second Experimental Group: Mice were divided into seven groups: Control group, Model group (3% DSS), Active Control (AC) group (Mesalazine, 0.39 g kg^−1^), PDA@MPD group (3.90 g kg^−1^), PDA group (0.076 g kg^−1^), MPD oral gavage group (I.g.-MPD, 3.12 g kg^−1^), and MPD enema group (En.-MPD, 3.120 g kg^−1^). Except for the Control group, UC models were induced by administering 3% DSS for 7 days, followed by sterile water for 3 days. Except for the Control and Model groups, all groups were administered the corresponding drugs via oral gavage or enema. Daily monitoring of body weight changes, fecal blood, and diarrhea was conducted. On day 8, mice were deeply anesthetized with sodium pentobarbital at 150 mg kg⁻^1^ (i.p.), subjected to retro-orbital bleeding, and immediately euthanized by cervical dislocation.

### In Vivo fluorescence imaging in mice

After a 24 h fast, mice received oral doses of FITC-MPD, PDA@FITC-MPD, or PDA@FITC-AB4. At 2 h, 6 h, and 24 h post-administration, in vivo fluorescence imaging was performed with a Kodak in vivo imaging system to monitor drug distribution within the mice. As a control, the Control group was given an equivalent volume of sterile water orally.

### Assessment of drug release from PDA@MPD

Considering the average gastric transit time of approximately 2 h, this study conducted a 2-h drug release experiment in simulated gastric fluid (pH 1.2) [19]. A solution of the PDA@MPD (10.0 mg mL⁻^1^, 5 mL) was placed in a dialysis bag (MWCO = 3,500 Da) and immersed in 200 mL of simulated gastric fluid (pH 6.0) in a 37℃ incubator with stirring at 60 rpm for 2 h. The dialysis bag was then transferred to 200 mL of simulated intestinal fluid for dialysis for an additional 48 h. Standard solutions at concentrations of 10, 20, 50, 100, and 200 µg mL⁻^1^ were prepared. The absorbance of each solution was measured. A standard curve was plotted with concentration on the x-axis and absorbance on the y-axis. Linear regression was performed to derive the regression equation. At predetermined time intervals, aliquots of the dialysate were collected, and the absorbance of AB4 and Ginsenoside RB1 was measured at wavelengths of 203 nm and 220 nm, respectively. Each time dialysate was collected, it was replaced with 5.0 mL of fresh dialysate. The release profiles of PDA@MPD at pH 1.2, 4.5, 6.0, and 7.4 were determined using the same protocol.

### Tissue sectioning and fluorescence detection

Mice were euthanized, and their colonic tissues were immersed in OCT (Optimal Cutting Temperature compound, SAKURA, USA) embedding medium and stored in a -80 ℃ freezer. Frozen sections of the small intestine tissue (10 µm thick) were prepared using a LEICA CM1900 cryostat (Leica, Germany). The frozen sections were stained with an antifade mounting medium containing DAPI (4',6-diamidino-2-phenylindole, Beyotime Biotechnology, China) to visualize nuclei and the fluorescence of the nanoparticles retained in the intestinal lumen. Fluorescence images of the tissue sections were acquired using an Olympus FV1200 confocal microscope (Olympus, Japan).

### Hematoxylin and eosin (H&E) staining

Mice were euthanized, and their colonic tissues were fixed overnight in 4% paraformaldehyde. After dehydration, the intestinal segments were embedded longitudinally in paraffin and then cut into 5 µm thick transverse sections for H&E staining. Representative sections were imaged using an Olympus inverted fluorescence microscope. Three mice from each treatment group were evaluated.

### Determination of active components by UV–vis spectroscopy

Standard and test solutions were prepared according to the reference method [20]. The contents of AB4, Ginsenoside RB1 and Militarine were measured using a SHIMADZU UV-2600i ultraviolet spectrophotometer. The detection wavelengths were 203 nm, 220 nm and 201 nm, respectively.

### Disease activity index (DAI)

Mice were daily weighed and monitored for fur condition, stool consistency, feeding behavior, activity, and mortality. DAI scores were calculated based on body weight loss, stool consistency, and fecal bleeding. Scoring criteria for body weight loss: < 1% loss: 0 points; 1%–5% loss: 1 point; 5%–11% loss: 2 points; 11%–15% loss: 3 points; > 15% loss: 4 points. For stool consistency: normal (formed, granular): 0 points; loose (pasty, non-adherent to the anus): 2 points; diarrhea (watery, adherent to the anus): 4 points. For fecal bleeding: normal: 0 points; occult blood (+): 2 points; bloody stool: 4 points. DAI score = (body weight loss score + stool consistency score + fecal bleeding score)/3 [21].

### Western blotting

Total proteins were extracted from colonic tissues at 4 ℃ using a total protein extraction kit. SDS-PAGE gels were prepared according to the instructions of the gel kit and the molecular weight of the target proteins. After adding 2 µL of colored marker (Novozyme Biotechnology Co., Ltd., China) to the edge well, total protein samples were loaded in the order of the experimental groups, separated by electrophoresis, and transferred to a PVDF membrane. The membrane was blocked with 5% Bovine Serum Albumin (BSA) at room temperature for 90 min to prevent nonspecific binding, followed by incubation with primary antibodies overnight at 4 ℃ with shaking at 50 rpm. The next day, after washing the primary antibodies, appropriate secondary antibodies were added and incubated at room temperature for 1 h. The membrane was then washed three times and imaged using a chemiluminescent imaging system.

### Enzyme-linked immunosorbnent assay (ELISA)

The levels of TNF-α, IL-1β, and IL-10 in mouse serum were measured according to the instructions of the ELISA kits.

### Quantitative reverse transcription polymerase chain reaction (qRT-PCR)

Total RNA was extracted from designated colonic samples using an RNA extraction kit and reverse-transcribed into cDNA using a Hiscript III RT SuperMix for qPCR (+ gDNA wiper) kit (Novozyme Biotechnology Co., Ltd., China). qRT-PCR was performed on the resulting cDNA using a Tag Pro Universal SYBR qPCR Master Mix, with β-actin used for the normalization of gene expression. Specific primer sequences are shown in Table 1.

**Table 1:** Primer sequences

Gene	Primer sequences (5′-3’)
F -mouse *IL-10*	GCTCTTACTGACTGGCATGAG
R -mouse *IL-10*	CGCAGCTCTAGGAGCATGTG
F -mouse *IL-6*	TAGTCCTTCCTACCCCAATTTCC
R -mouse *IL-6*	TTGGTCCTTAGCCACTCCTTC
F -mouse *IL-1β*	GCAACTGTTCCTGAACTCAACT
R -mouse *IL-1β*	ATCTTTTGGGGTCCGTCAACT
F -mouse *TNF-α*	CCCTCACACTCAGATCATCTTCT
R -mouse *TNF-α*	GCTACGACGTGGGCTACAG
F -mouse *Occludin*	TAAGAGCTTACAGGCAGAACTAG
R -mouse *Occludin*	CTGTCATAATCTCCCACCATC
F -mouse *ZO-1*	GCCGCTAAGAGCACAGCAA
R -mouse *ZO-1*	TCCCCACTCTGAAAATGAGGA
F -mouse *β-actin*	ATGACCCAAGCCGAGAAGG
R -mouse *β-actin*	CGGCCAAGTCTTAGAGTTGTTG

### Statistical analysis

One-way ANOVA was used to evaluate the statistical significance among experimental groups. #: *P* < 0.05, vs Model group or MPD group. *: *P* < 0.05, **: *P* < 0.01, ***: *P* < 0.001, ****: *P* < 0.0001, vs Model group or MPD group. #*: *P* < 0.05, vs En.-MPD group. ns = not significant, vs Model or MPD group. Unless otherwise specified, all data are presented as mean ± SEM.

## Results

### Synthesis and characterization of PDA@MPD

**Fig. 1:**
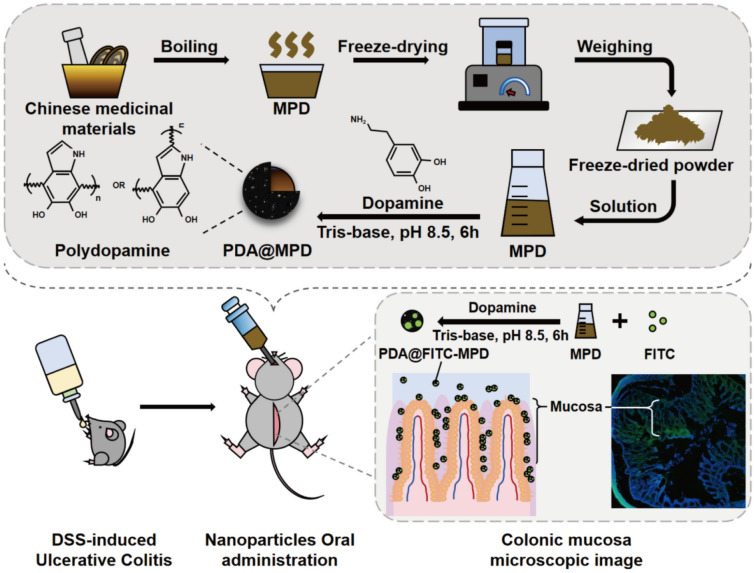
Graphical abstract. An oral traditional Chinese medicine–based nanoparticle formulation for DSS-induced ulcerative colitis in mice. Designed to prolong colonic residence

The preparation of PDA-encapsulated MPD nanoparticles is outlined in the graphical abstract (Fig. 1). Briefly, the Chinese medicinal herbs mixture used for MPD was decocted, concentrated and freeze-dried to yield MPD lyophilised powder, which served as the core material. This powder was immersed in an alkaline dopamine solution, where oxidation and self-polymerization occurred to produce PDA. As PDA nucleation begins at the solid–liquid interface, the resultant coating deposits on the MPD core surface, yielding core–shell nanoparticles.

**Fig. 2:**
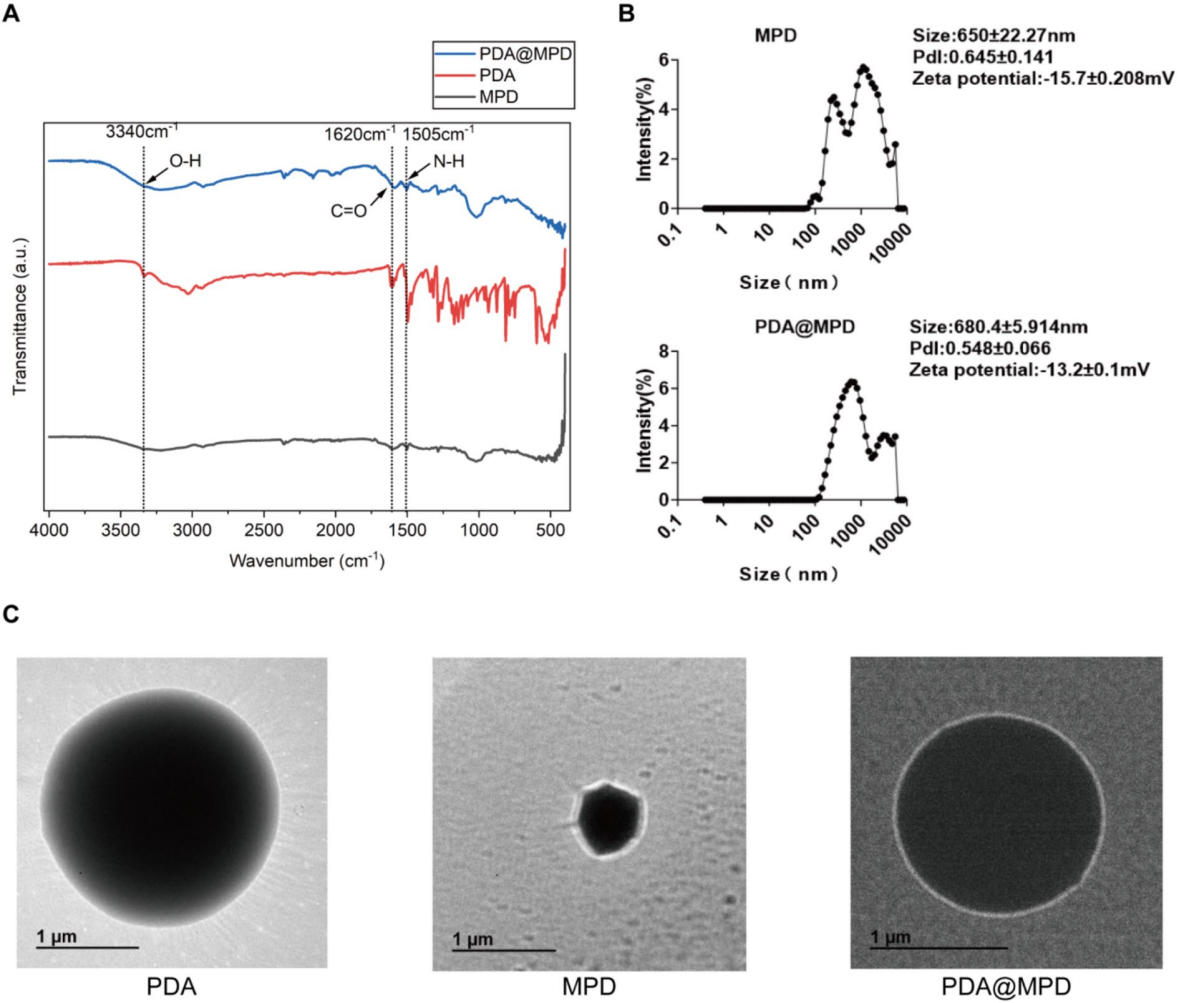
Characterization of PDA@MPD. **A** FT-IR spectra of PDA@MPD, PDA and MPD. **B** The hydrodynamic particle size and surface charge of PDA@MPD and MPD. **C** Transmission electron micrograph of PDA, MDP and PDA@MPD. Mean ± SEM, n = 3 replicates

Subsequently, Fourier Transform Infrared Spectroscopy (FT-IR) was used to characterize the surface chemical structure of PDA@MPD to assess the presence of PDA on the nanoparticle surface. The results showed three characteristic peaks at wavenumbers of 1505, 1620, and 3340 cm⁻^1^, consistent with PDA (Fig. 2A). The hydrodynamic particle size of PDA@MPD was measured using a Malvern Zetasizer Model-Nano ZS. After PDA coating, the mean diameter increased slightly, and the error range narrowed, indicating a more homogeneous system (Fig. 2B). The morphology and particle size distribution of PDA@MPD were characterized using transmission electron microscopy (TEM). Under the microscope, PDA@MPD appeared as spherical granules. The particle size was estimated at approximately 2.0 µm under TEM (Fig. 2C), TEM-derived particle sizes may exceed the hydrodynamic diameters measured by the Malvern Zetasizer Nano ZS, depending on the extent of particle aggregation and the nature of surface modifications. These results support PDA modification of MPD.

### PDA@MPD colonic retention

**Fig. 3:**
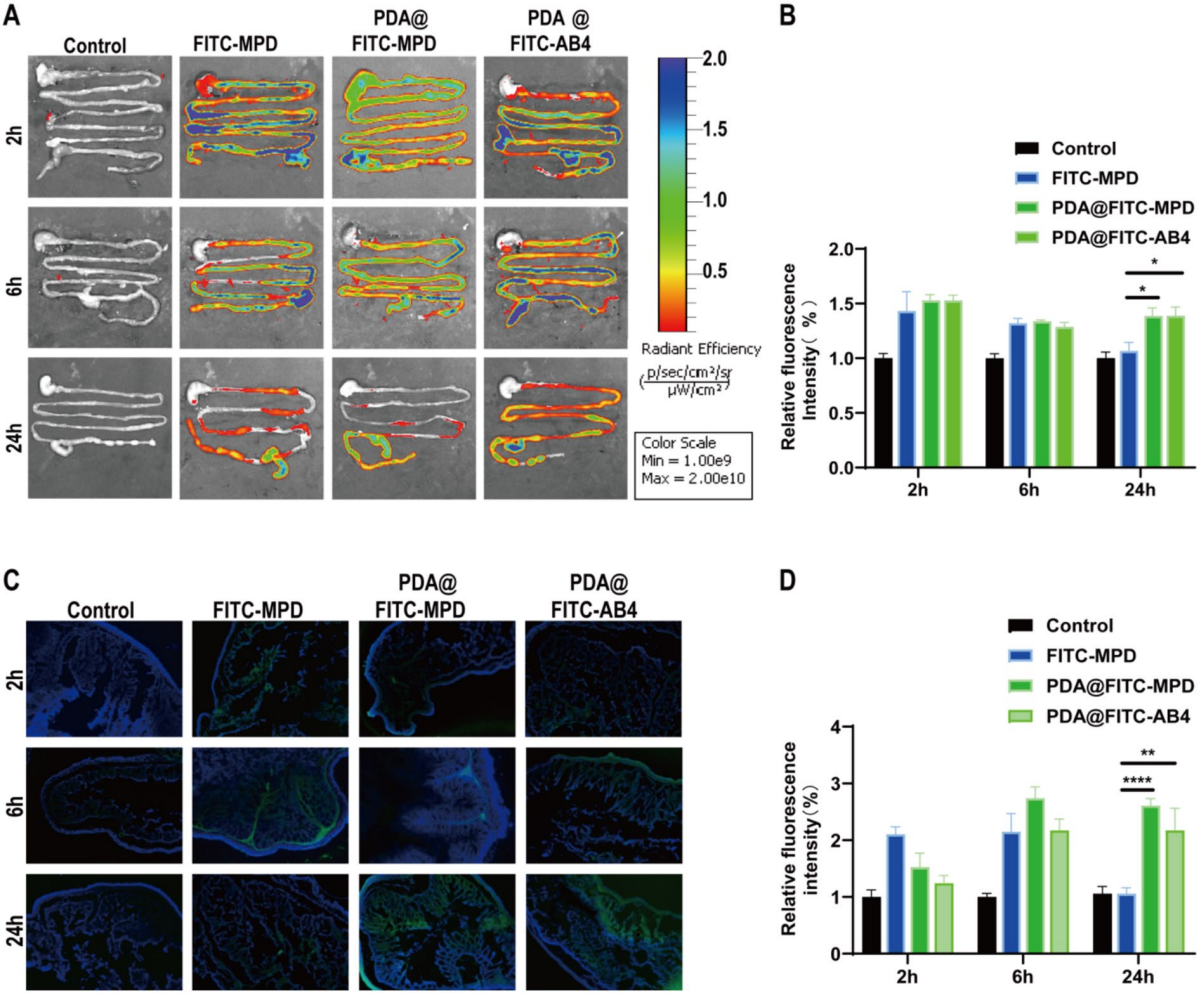
Retention of PDA@MPD in the colon. **A** Representative in vivo images illustrating the distribution of label-free PDA@MPD, FITC-MPD, PDA@FITC-MPD and PDA@FITC-AB4 in colon at 2 h, 6 h and 24 h after oral administration. **B**&**D** Changes of FITC fluorescence intensity for 2 h, 6 h and 24 h after oral administration of various drugs. **C** Distribution of green fluorescent FITC-MPD, PDA@FITC-MPD and PDA@FITC-AB4 in transverse cryosections of mice colons. Scale bars: 200 μm. Data are mean ± SEM, n = 3. *: *P* < 0.05, **: *P* < 0.01, ****: *P* < 0.0001, vs FITC-MPD group

To investigate the colon retention of PDA@MPD, we used FITC, a fluorescent agent that can covalently bind to various molecules, to label MPD for intestinal fluorescence imaging to track the distribution of PDA@MPD at different time points. Since MPD is a compound drug containing multiple active ingredients, one of the main active components is AB4, and FITC has good binding activity with AB4 (Supplementary Fig. 1). Therefore, we also labeled the single herbal component AB4 with FITC as a control. After labeling, we encapsulated them with PDA using the same method and studied their retention characteristics in the mouse gastrointestinal tract. In vivo fluorescence imaging of the mouse gastrointestinal tract was performed at 2 h, 6 h, and 24 h after administration (Fig. 3A-B). The results showed that, FITC-MPD, PDA@FITC-MPD, and PDA@FITC-AB4 all began to enter the colon at 2 h after oral administration and reached maximal colonic filling by 6 h. However, compared with FITC-MPD, PDA@FITC-MPD and PDA@FITC-AB4 maintained relatively strong fluorescence signals in the colon at 24 h after oral administration. Furthermore, colonic cryosections were prepared to localise fluorescence within the mucosa (Fig. 3C-D). Compared with the Control group without green fluorescence, FITC-MPD exhibited strong fluorescence in the colon at 2 h after administration, which almost disappeared at 24 h due to the flow and clearance effects of the colonic mucus layer and digestive fluids. In contrast, PDA-coated particles accumulated along the crypt bases and retained readily detectable fluorescence at 24 h. This retention is likely due to the adhesive properties of polydopamine, which resist digestive flushing. Collectively, PDA encapsulation prolonged colonic retention of MPD in mice.

**Fig. 4:**
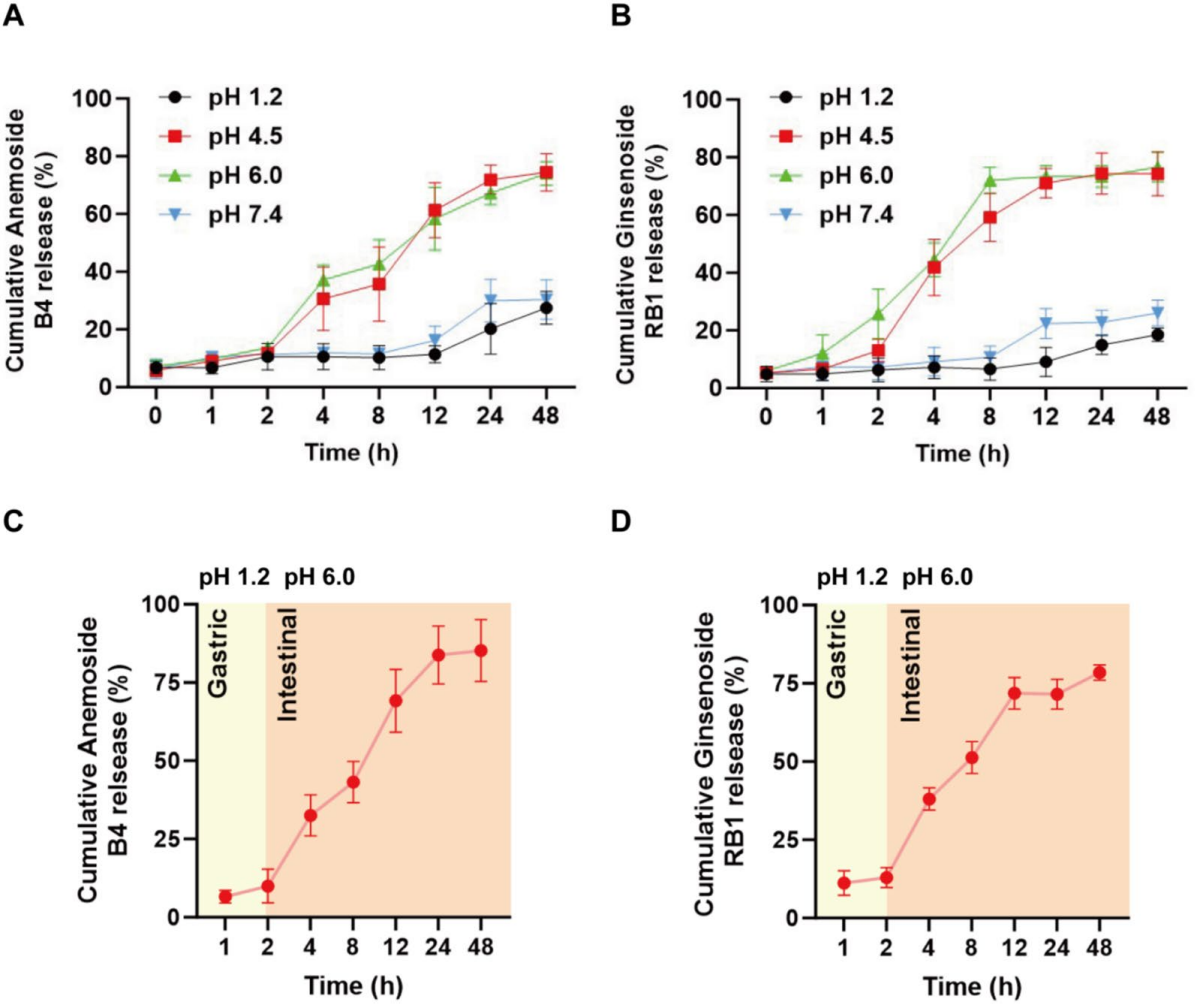
In vitro drug release profiles of PDA@MPD: (**A**, **B**) at different pH values; (**C**, **D**) in simulated gastrointestinal environments. Mean ± SEM, n = 3 replicates

PDA can regulate drug release via pH sensitivity [12, 13]. The PDA coating exhibits greater stability at pH 1.2 (gastric) and pH 7.4 (physiological), whereas gradual disintegration occurs at pH 4.5–6.0 (intestinal), facilitating cargo release (Fig. 4A, B). To evaluate the release profile, PDA@MPD was incubated in simulated gastric fluid (SGF, pH 1.2, 2 h) and simulated intestinal fluid (SIF, pH 6.0, 48 h). In SGF, the active components (AB4 and Ginsenoside RB1) of PDA@MPD exhibited a suppressed drug release profile and good stability, likely due to the polydopamine coating that resists gastric acid erosion (Fig. 4C, D). In contrast, in SIF, the active components were gradually released from the PDA nanocarriers, suggesting sustained-release kinetics in the intestinal environment.

**Fig. 5:**
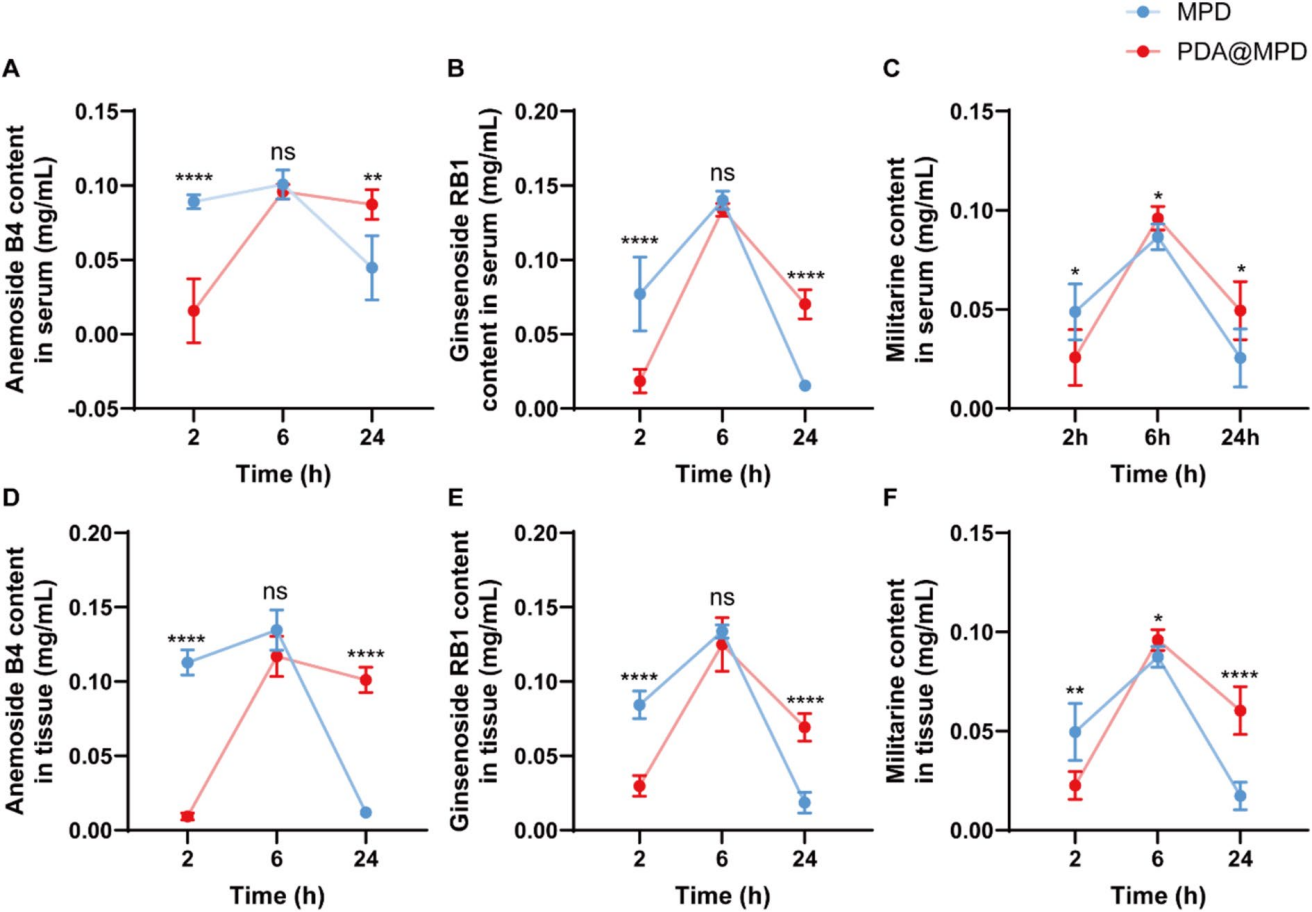
Determination of active components by UV–vis spectroscopy. **A**-**F** Quantification of Anemoside B4, Ginsenoside RB1, and Militarine in serum and colon tissue at 2 h, 6 h, and 24 h post PDA@MPD administration. Data are mean ± SEM. n = 3 replicates. *P < 0.05, **P < 0.01, ***P < 0.001, ****P < 0.0001 vs MPD; ns = not significant vs MPD

To further evaluate the prolonged residence of PDA@MPD, we measured the active components (AB4, Ginsenoside RB1, and Militarine) in mouse blood and colon tissue. As shown in Fig. 5A-F, compared to the MPD group, the PDA@MPD group demonstrated higher levels of active components in serum and tissue samples at 24 h. These data suggest that PDA coating prolonged the residence of MPD-derived constituents in vivo.

### PDA@MPD effects on DSS-induced colonic damage in mice

**Fig. 6:**
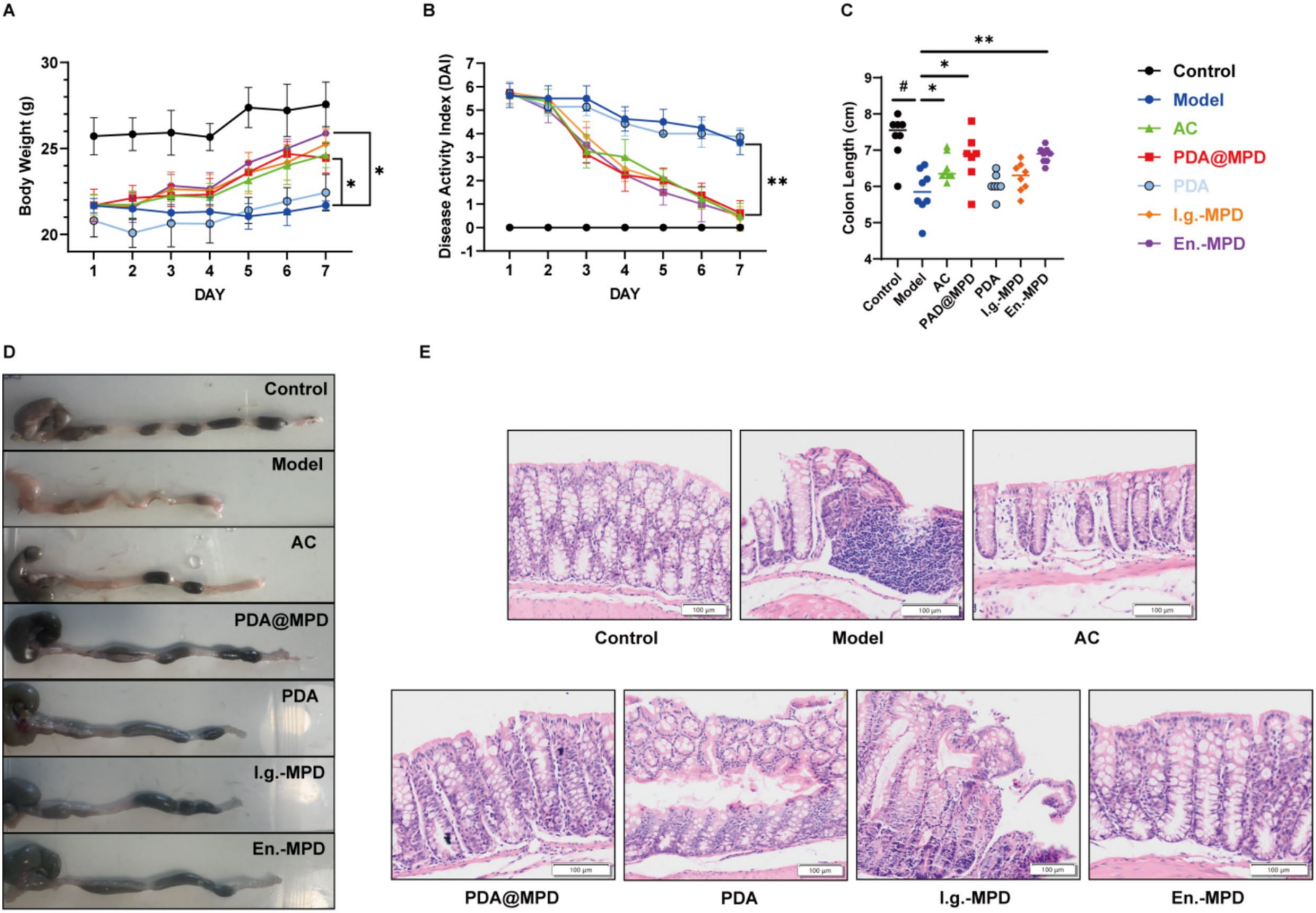
PDA@MPD reduced DSS-induced colonic damage. **A** Body weight change. **B** DAI scores. (**C**&**D**) Length of colons. **E** H&E staining of colonic tissue (200 ×). Data are mean ± SEM, n = 6–8. #: *P* < 0.05, vs Model group. *: *P* < 0.05, **: *P* < 0.01, vs Model group

LC–MS analyses in our earlier work identified marker constituents of MPD and provided preliminary pharmacological data [22, 23]. Here, the anti-colitic activity of PDA@MPD was examined in DSS-induced ulcerative colitis mice. Compared with the Control group, the body weight of mice in all other groups significantly decreased after modeling. Over the subsequent 7-day treatment period, weight changes differed among groups. Weight recovery was more pronounced in PDA@MPD and En.-MPD groups versus Model mice (*P* < 0.05) (Fig. 6A). Additionally, except for the Model and PDA groups, DAI scores of DSS-induced UC mice markedly decreased after treatment (*P* < 0.01) (Fig. 6B), while the Control group remained unchanged. Colon shortening in Model mice confirmed mucosal atrophy (*P* < 0.05). Treatment with mesalazine (AC group) attenuated this shortening, although full restoration to Control levels was not achieved (*P* < 0.05). Both the PDA@MPD and En.-MPD groups showed improvement (*P* < 0.05), with effects comparable to AC. A modest—but statistically non-significant—amelioration was observed in the I.g.-MPD group (*P* > 0.05), whereas PDA alone showed no significant effect (*P* > 0.05). Collectively, PDA@MPD, by virtue of targeted MPD delivery, outperformed conventional intragastric administration (I.g.-MPD), however, it did not surpass the efficacy of En.-MPD, a finding that may reflect substantial inter-individual variability within the PDA@MPD cohort. Mesalazine (AC) served as a positive control and validated model reliability, yet both the nanoformulation and enema-based delivery exhibited superior trends. (Fig. 6C-E). These data indicate that PDA@MPD reduces DSS-induced colonic injury in mice, with modest effects from unmodified MPD.

### PDA@MPD effects on DSS-induced intestinal inflammation in mice

**Fig. 7:**
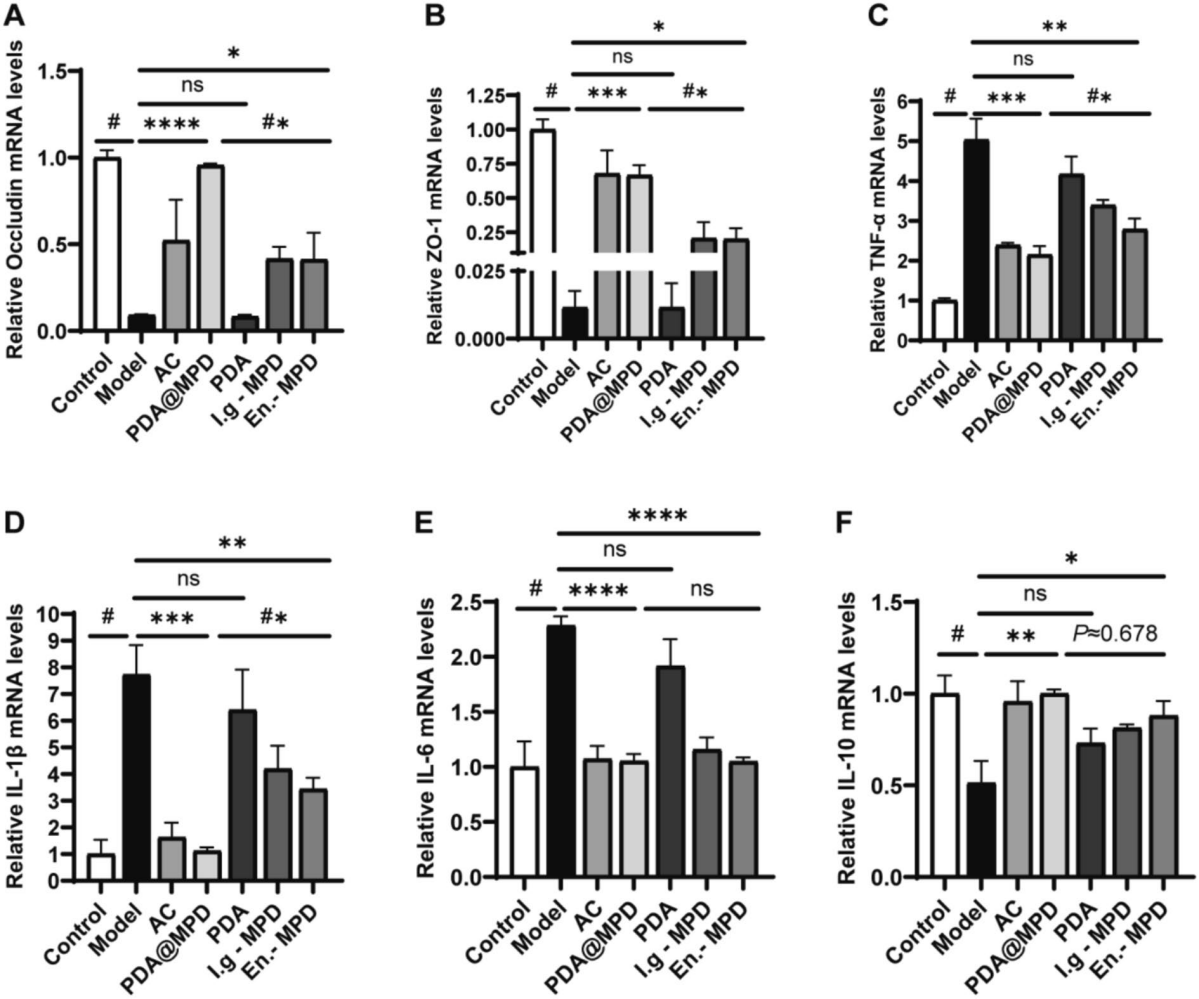
PDA@MPD promote the repair of the intestinal barrier and reduce DSS-induced intestinal inflammation. **A**–**F** Relative mRNA expression levels of Occludin, ZO-1, TNF-α, IL-1β, IL-6 and IL-10. Data are mean ± SEM, n = 4. #: *P* < 0.05, vs Model group. *: *P* < 0.05, **: *P* < 0.01, ***: *P* < 0.001, ****: *P* < 0.0001, vs Model group. #*: *P* < 0.05, vs En.-MPD group. ns = not significant, vs Model or En.-MPD group

**Fig. 8:**
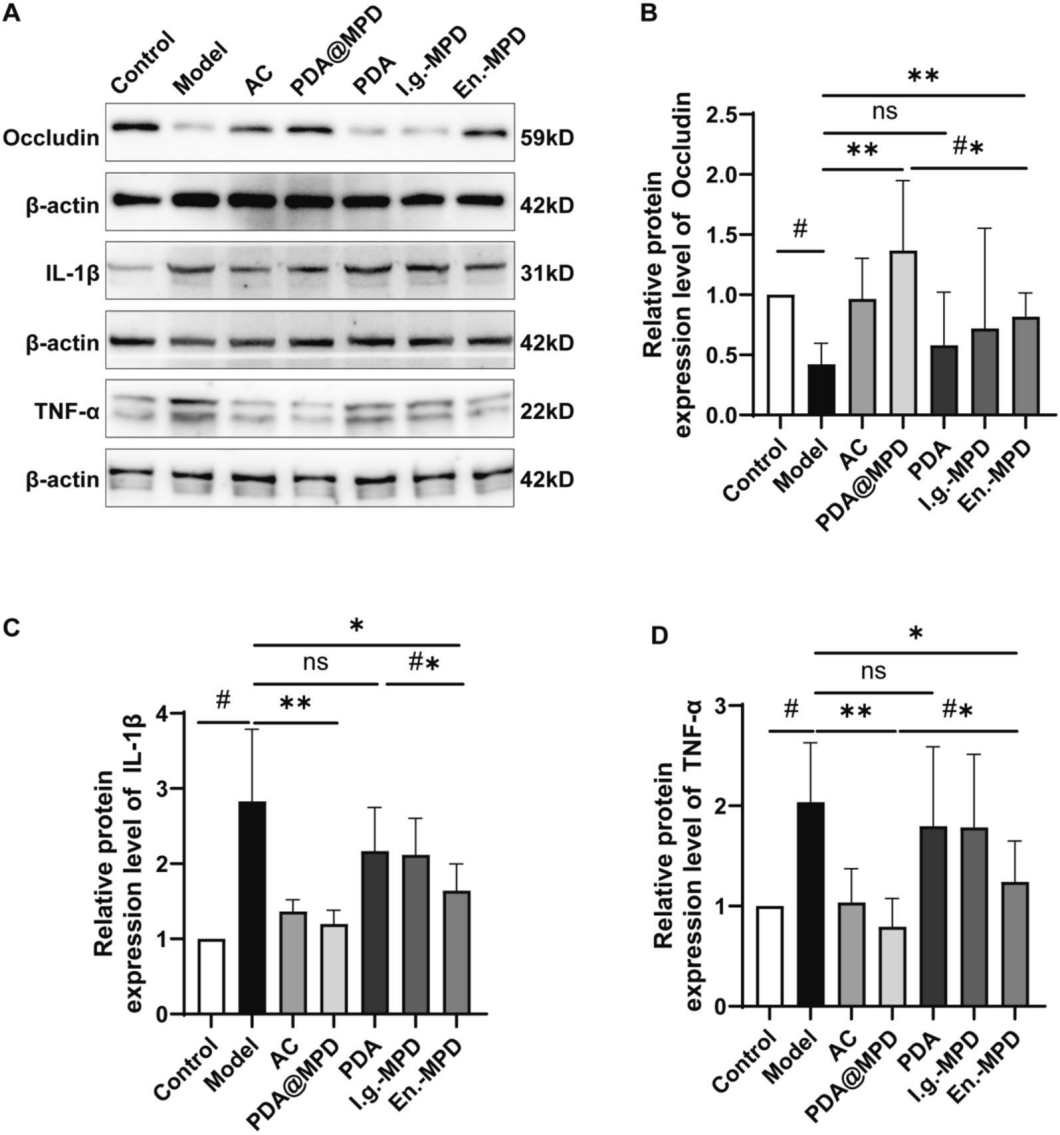
PDA@MPD increase the expression levels of tight junction protein and reduce the expression levels of pro-inflammatory cytokines. **A**–**D** Protein expression levels of Occludin, TNF-α, and IL-1β evaluated by WB. Data are mean ± SEM, n = 8. #: *P* < 0.05, vs Model group. *: *P* < 0.05, **: *P* < 0.01, ***: *P* < 0.001, vs Model group. #*: *P* < 0.05, vs En.-MPD group. ns = not significant, vs Model group

**Fig. 9:**
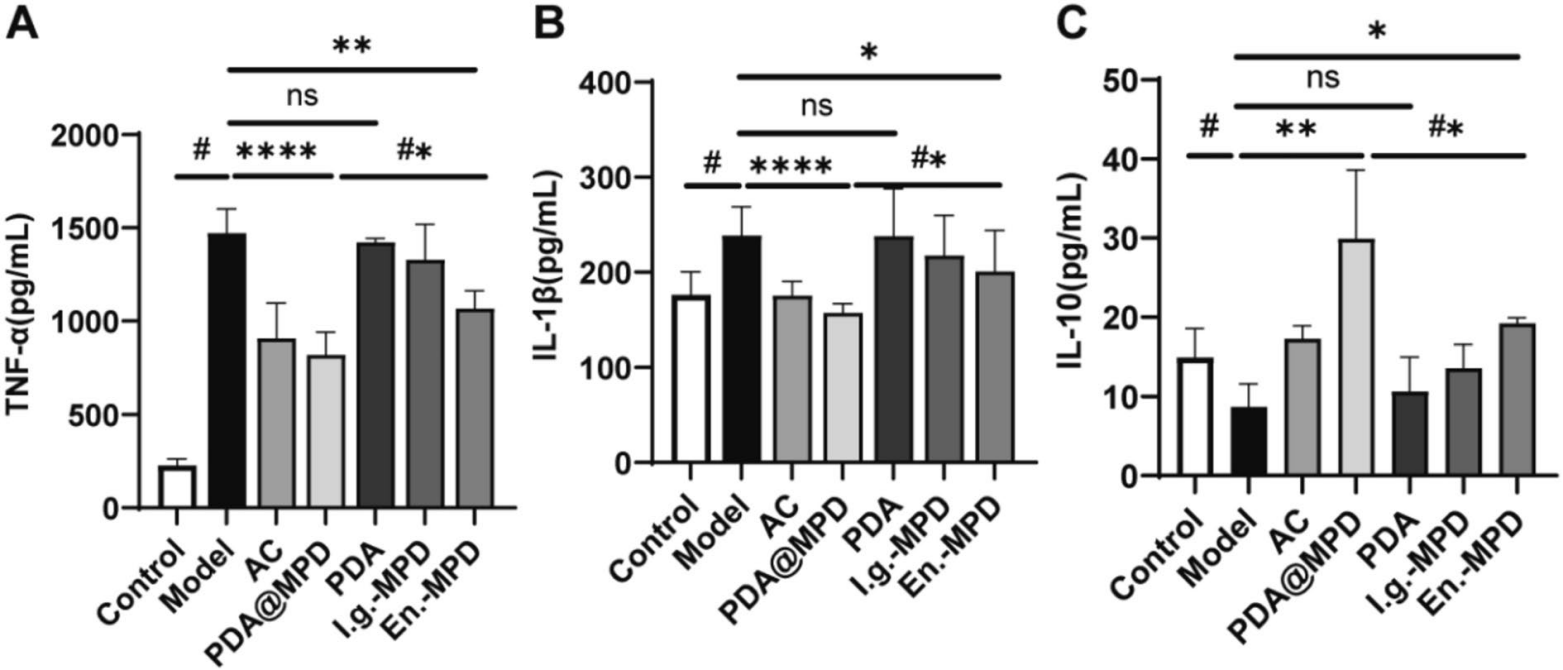
Regulation of inflammatory cytokine production by PDA@MPD. **A**–**C** Serum TNF-α, IL-1β and IL-10 levels. Data are mean ± SEM, n = 8. #: *P* < 0.05, vs Model group. *: *P* < 0.05, **: *P* < 0.01, ****: *P* < 0.0001, vs Model group. #*: *P* < 0.05, vs En.-MPD group. ns = not significant, vs Model

To further explore the therapeutic effects of PDA@MPD on DSS-induced UC, we conducted experimental tests on biological samples from mice in each group. The results showed that, in terms of intestinal barrier function, compared to the Model group, the relative mRNA expression levels of Occludin and Zonula Occludens-1 (ZO-1) in the colon tissues of PDA@MPD and En.-MPD group mice were upregulated (Fig. 7A-B), and the protein expression levels of Occludin also showed an upward trend (Fig. 8A-B). Regarding inflammatory responses, compared to the Model group, the relative mRNA expression levels of TNF-α, IL-1β, and IL-6 were downregulated in the PDA@MPD and En.-MPD groups, while the relative mRNA expression levels of IL-10 were upregulated (Fig. 7C-F). Consistently, the protein expression levels of TNF-α and IL-1β in mouse colon tissues were downregulated (Fig. 8A, C, D). Additionally, serum inflammatory factor levels were examined, compared to the Model group, the TNF-α and IL-1β levels were reduced, while IL-10 levels were upregulated in the PDA@MPD and En.-MPD groups (Fig. 9A-C). Notably, compared with the En.-MPD group, the PDA@MPD group showed more significant improvements. Collectively, the data indicate that both PDA@MPD and MPD promote the repair of the intestinal barrier and reduce DSS-induced intestinal inflammation, with PDA@MPD demonstrating superior therapeutic effects to MPD.

## Discussion

Ulcerative colitis (UC) is a superficial, diffuse and continuous inflammatory disease of the colonic mucosa that typically presents with diarrhoea, abdominal pain, rectal bleeding and weight loss [24]. Its recurrent and refractory course represents a major clinical challenge [25]. Over the past three decades, its incidence has increased worldwide, adding considerable economic burden to health-care systems and to patients and their families. UC pathogenesis is associated with dysfunction of the intestinal mucosal mechanical barrier, which comprises a cellular and a mucus component [26]. The cellular component consists mainly of columnar epithelial cells interspersed with goblet, Paneth and neuroendocrine cells, and is sealed by tight junctions (TJs) [27]. TJs play a crucial role in regulating the selective permeability of the intestinal epithelial barrier, maintaining mucosal homeostasis, and preventing the entry of pathogenic antigens from the intestinal lumen into the mucosal lamina propria, which could otherwise trigger intestinal and systemic inflammatory and immune responses [28]. These junctions comprise transmembrane proteins, peripheral membrane proteins and claudins. Occludin is a key transmembrane protein of TJs, its abundance correlates with barrier integrity [29, 30]. The peripheral membrane zonula occludens (ZO) family (ZO-1, ZO-2 and ZO-3) links transmembrane TJ proteins to the cytoskeleton. In DSS-induced colitis, reduced ZO-1 expression and increased epithelial permeability precede overt inflammation, implying that TJ dysfunction may contribute to disease initiation [31]. Therefore, restoration of the mucosal mechanical barrier therefore represents a rational therapeutic target in UC.

Traditional Chinese medicine (TCM) considers Pulsatilla Decoction beneficial in managing UC. Modern pharmacological studies report that Pulsatilla Decoction's main chemical components, such as AB4 and Luteolin, have anti-inflammatory, antioxidant, antibacterial, and wound-healing properties. The *2022 Integrative Medicine Expert Consensus on Ulcerative Colitis Diagnosis and Treatment* and *2023 World Federation of Chinese Medicine Societies Digestive System Disease Specialty Committee International Clinical Practice Guideline for Traditional Chinese Medicine in Ulcerative Colitis* emphasize the importance of TCM in UC treatment strategies and recommend modifying the core formula based on the condition [32, 33]. Accordingly, we developed MPD. Our previous studies showed that MPD increased Occludin and ZO-1 protein expression by inhibiting the p38 MAPK/MLCK pathway, reduces serum endotoxin levels, promotes intestinal mucosal barrier repair, maintains tight junction integrity, and decreases intestinal mucosal permeability, thereby treating UC [10, 23]. Despite these benefits, poor patient compliance, the need for trained personnel to perform enemas, and short colonic retention remain obstacles to wider clinical use, highlighting the need for alternative delivery strategies.

Dopamine, the primary secretory component of marine mussel byssal threads, adheres to many materials in seawater [34]. This water-resistant adhesion may facilitate MPD retention along the fluid-filled intestinal lumen, thereby increasing colonic accumulation and residence time [14, 15]. In alkaline conditions, dopamine's catechol group oxidizes to dopamine quinone [35]. Dopamine quinone undergoes intramolecular cyclization to form an intermediate structure, which is then oxidized to form an unstable 5,6-dihydroxyindole intermediate. This intermediate undergoes intermolecular and intramolecular rearrangements and is further oxidized to form polydopamine [36]. Polydopamine, with its excellent acid resistance, retention, and biocompatibility, has been widely studied in the scientific community [16].

Building on existing data, we sought to overcome the limitations of MPD retention enemas and to prolong colonic drug exposure. We therefore formulated an oral PDA-encapsulated MPD nanoparticle that exploits polydopamine's adhesive and pH-responsive properties. This nanoparticle provides sustained cargo release and extended colonic residence, potentially improving local therapeutic levels of MPD.

FITC is a fluorescent dye that can be used to label a variety of biomolecules through covalent coupling with amines, hydroxyl groups, and other functional groups [37]. In this study, FITC was used to label the MPD and nanoparticle to observe its retention in mice. The results showed that fluorescence accumulated along the colonic mucosa at 6 h and remained detectable at 24 h. Compared with the FITC-MPD group, the retention time of PDA@FITC-MPD in the intestine was significantly prolonged. These data indicate that PDA encapsulation delivers MPD to the colon and prolongs its local residence, potentially increasing colonic drug levels.

To evaluate the anti-colitic activity of MPD, we induced acute colitis with 3% DSS in C57BL/6 mice, a model that reproduces key clinical features of human UC. After treatment with PDA@MPD, compared with the MPD group, PDA@MPD showed a more pronounced inhibitory effect on DSS-induced colonic damage. This improvement was accompanied by elevated tight-junction proteins (Occludin, ZO-1), reduced pro-inflammatory cytokines (TNF-α, IL-1β) and increased IL-10. Polydopamine prolonged colonic residence of MPD, which may contribute to the observed molecular and histological improvements. This nanoparticle may offer insights for enhancing local drug delivery to other gastrointestinal segments while potentially minimising systemic exposure.

This study provides proof-of-concept that polydopamine encapsulation can be used to deliver a traditional Chinese medicine formula to the colon. However, several limitations should be acknowledged. First, release profiles were characterised only in simulated gastrointestinal fluids; comprehensive in vivo pharmacokinetics remain to be investigated. Second, the molecular basis of PDA adhesion to colonic mucosa and the downstream therapeutic pathways of PDA@MPD require further elucidation.

## Conclusion

In summary, we developed an oral nanoparticle formulation in which polydopamine encapsulates the modified traditional Chinese medicine formula MPD. The nanoparticle formulation enables oral delivery, prolongs colonic residence and shows sustained release characteristics. This approach shifts MPD from rectal enema to oral administration and increases local colonic drug levels. Our findings provide a proof-of-concept for oral TCM delivery in UC and may offer insights for local drug targeting to other intestinal segments.

## Supplementary Information

Supplementary file 1.

## Data Availability

No datasets were generated or analysed during the current study.

## Abbreviations

PDA: Polydopamine
MPD: Modified Pulsatilla decoction
UC: Ulcerative colitis
TCM: Traditional Chinese medicine
FT-IR: Fourier transform infrared spectroscopy
FITC: Fluorescein isothiocyanate
DSS: Dextran sulfate sodium
DAI: Disease activity index
IBD: Inflammatory bowel disease
AB4: Anemoside B4
IL-1β: Interleukin-1 beta
TNF-α: Tumor necrosis factor alpha
IL-10: Interleukin-10
IL-6: Interleukin-6
AC: Active control
OCT: Optimal cutting temperature
BSA: Bovine serum albumin
ELISA: Enzyme-linked immunosorbnent assay
qRT-PCR: Quantitative reverse transcription polymerase chain reaction
ZO-1: Zonula occludens-1
TEM: Transmission electron microscopy
TJ: Tight junctions
