# Supplementary material for: Polydopamine-encapsulated modified Pulsatilla decoction: a strategy to enhance ulcerative colitis therapy
Source: Chin Med. 2026 Jan 5;21:2. doi: 10.1186/s13020-025-01280-1 (PMC12766960; doi:10.1186/s13020-025-01280-1)
Supplement: Supplementary file 1 [file 13020_2025_1280_MOESM1_ESM.docx]

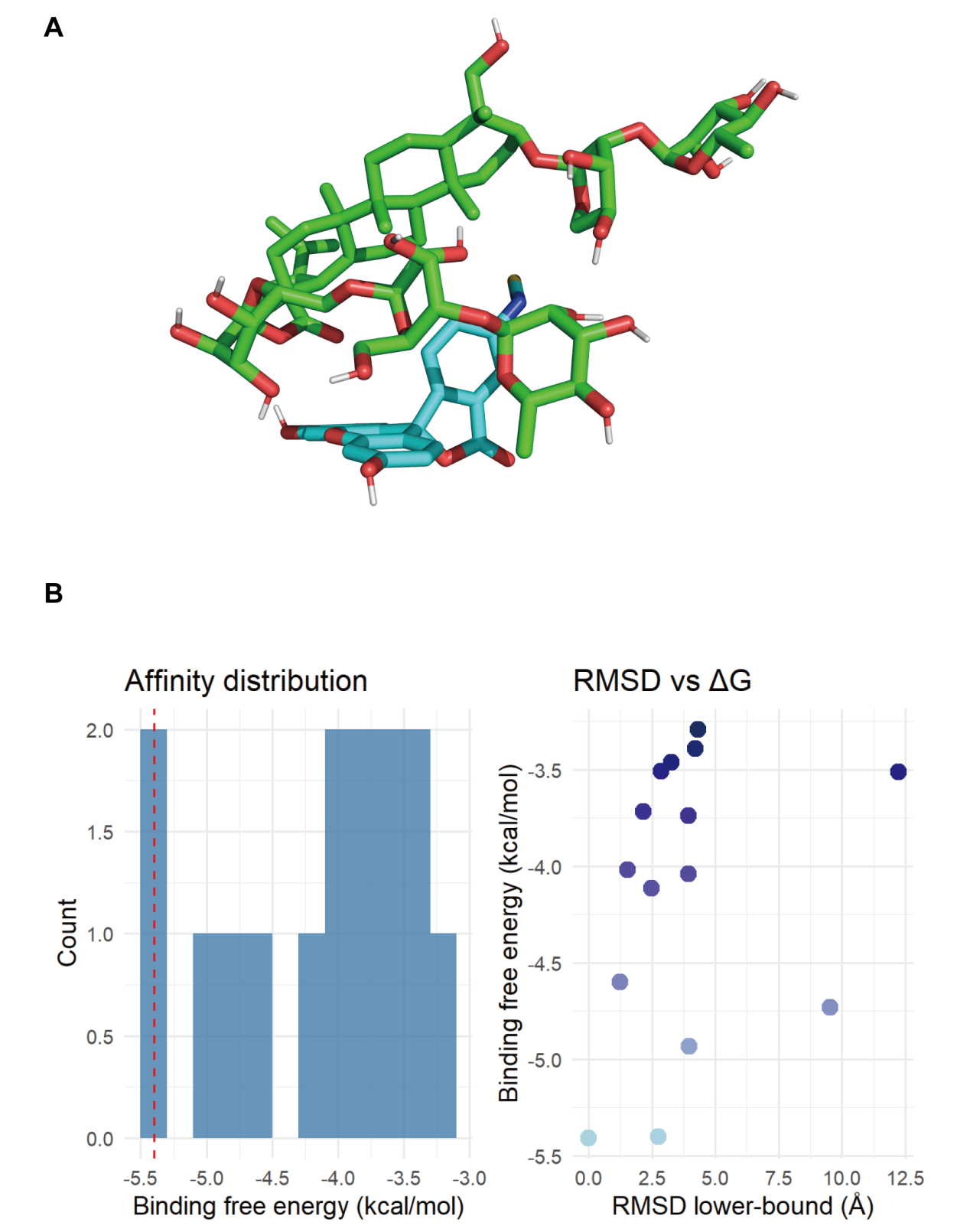


**Supplementary Fig. 1.** Molecular Docking Results of FITC with AB4. (A&B) Scatter plots depict root-mean-square deviation (RMSD, lower-bound, Å) versus binding free energy (ΔG, kcal mol⁻¹) for the FITC–AB4 complex. Data span ΔG from –5.0 to 0 kcal mol⁻¹ and RMSD from 0 to 5.5 Å. Two dense clusters at ΔG ≈ –4.5 to –3.5 kcal mol⁻¹ and RMSD ≈ 1.0–2.5 Å indicate energetically similar, low-RMSD binding modes, confirming weak-to-moderate affinity and structural stability.
